# Reused Cultivation Water Accumulates Dissolved Organic Carbon and Uniquely Influences Different Marine Microalgae

**DOI:** 10.3389/fbioe.2019.00101

**Published:** 2019-05-14

**Authors:** Sarah E. Loftus, Zackary I. Johnson

**Affiliations:** ^1^Duke University Marine Lab, Nicholas School of the Environment, Beaufort, NC, United States; ^2^Department of Biology, Trinity College of Arts and Sciences, Duke University, Durham, NC, United States

**Keywords:** algae cultivation, water reuse, dissolved organic carbon, algae biotechnology, bacteria, marine microalgae, recalcitrance

## Abstract

Reusing growth medium (water supplemented with nutrients) for microalgae cultivation is required for economical and environmentally sustainable production of algae bioproducts (fuels, feed, and food). However, reused medium often contains microbes and dissolved organic matter that may affect algae growth. While the accumulation of dissolved organic carbon (DOC) in reused medium has been demonstrated, it is unclear whether DOC concentrations affect algae growth or subsequent rates of algal DOC release. To address these questions, lab-scale experiments were conducted with three marine microalgae strains, *Navicula* sp. SFP, *Staurosira* sp. C323, and *Chlorella* sp. D046, grown in medium reused up to four times. *Navicula* sp. and *Chlorella* sp. grew similarly in reused medium as in fresh medium, while *Staurosira* sp. became completely inhibited in reused medium. Across the three algae, there was no broad trend between initial DOC concentration in reused medium and algae growth response. *Navicula* sp. released less DOC overall in reused medium than in fresh medium, but DOC release rates did not decrease proportionally with increased DOC concentrations. Net DOC accumulation was much lower than gross DOC released by *Navicula* sp. and *Staurosira* sp., indicating the majority of released DOC was degraded. Additionally, biodegradation experiments with reused media showed no further net decrease in DOC, suggesting the accumulated DOC was recalcitrant to the associated bacteria. Overall, these results suggest that taxa-specific factors may be responsible for algae growth response in reused medium, and that DOC release and accumulation are insensitive to prior cultivation rounds. Choosing an algae strain that is uninhibited by accumulated DOC is therefore critical to ensure successful water reuse in the algae industry.

## Introduction

Minimizing algae cultivation costs and maximizing algae productivity are priorities for producing algal bioproducts such as fuels, feed, and food. Reusing growth medium (water supplemented with nutrients) after algae harvesting is one crucial strategy to reduce costs (Farooq et al., 2015). However, reused medium may affect algae yields because it can contain dissolved compounds, cell debris, microbes, and any substances used in the harvesting process. While medium has been reused successfully in large-scale open ponds (White and Ryan, 2015), maximizing water reuse at low capital and operating costs is an active research area in the algae industry (U.S. DOE, 2016).

Previous studies have tested how reused medium affects algae growth using a variety of algae taxa, harvesting methods, and growth conditions. These studies revealed diverse growth responses ranging from inhibition to stimulation, though most experiments found no substantial effects caused by reused medium (Loftus and Johnson, 2017). One factor that accounted for the wide variety of growth responses was algae taxa.

Taxa-specific growth results in reused medium might be explained by dissolved organic matter released by algae. Microalgae release dissolved organic carbon (DOC) compounds during growth and decay, as well as when cells lyse. The concentration and composition of DOC depends on algae taxa (Henderson et al., 2008; Becker et al., 2014), growth conditions (Wear et al., 2015; Saad et al., 2016; Wu et al., 2016), growth phase (Barofsky et al., 2009), and associated microbes (Grossart and Simon, 2007). Algae can also actively release extracellular compounds to attach to or move across surfaces (Molino and Wetherbee, 2008). DOC accumulates in reused medium if it is recalcitrant to degradation by bacteria (as most algae grown for commercial purposes are not axenic). DOC lability is influenced by a combination of factors such as molecular weight and composition, nutrient concentrations, and metabolic potential of the bacteria community, among other factors (Amon and Benner, 1996; Sun et al., 1997; Carlson et al., 2004; Elifantz et al., 2007; Nelson and Carlson, 2012; Vorobev et al., 2018).

Understanding the dynamics of DOC composition and concentration in reused medium, as well as its effects on algae growth, is therefore necessary to optimize algae growth. Some types of DOC are known to be inhibitory to algae, such as fatty acids (McCracken et al., 1980; Ikawa et al., 1997; Bosma et al., 2008), which impair plasma membranes (Wu et al., 2006). DOC build-up can also affect chemical and physical properties of the water, which can affect gas transfer (Filali Mouhim et al., 1993) and cell aggregation (Zhang et al., 2016).

While numerous studies have measured dissolved organic compounds in reused medium experiments (e.g., Hadj-Romdhane et al., 2013; Depraetere et al., 2015; Zhang et al., 2016), few have correlated DOC concentrations in reused medium with algae growth response. For example, some studies found that increased concentrations of DOC or certain fractions of DOC promoted algae growth, likely because the algae were mixotrophic (Burkiewicz and Synak, 1996; Fon Sing et al., 2014). Another study diluted reused medium and found that higher DOC concentrations were more inhibitory (Zhang et al., 2013). Depraetere et al. (2015) found that a relatively large size-fraction (>3 kDa) of reused medium was mildly growth-inhibitory compared to greater inhibition observed in unfiltered medium, indicating that smaller molecules were mostly responsible. Studies have also shown that removal or degradation of DOC, via methods such as activated carbon absorption (Zhang et al., 2016) and advanced oxidation processes (Wang et al., 2018), eliminated growth inhibition in reused medium. However, to our knowledge no studies have determined if the DOC concentration in reused medium broadly correlates with algae growth response across physiologically different algae taxa. Additionally, studies have not addressed whether accumulated DOC in reused medium affects subsequent algal DOC release rates.

The objectives of this study were therefore to (a) characterize growth responses of microalgae strains in reused medium, (b) measure gross DOC excretion and net DOC accumulation over multiple medium reuses, and (c) determine if DOC concentration in reused medium is associated with algae growth response. To check bioavailability of the accumulated DOC, reused media were also used to investigate long-term DOC degradation by residual bacteria. Understanding growth responses and DOC release patterns across different algae taxa could help predict growth success and DOC accumulation in reused medium, and inform algae selection and harvesting schedules to maximize biomass yields.

## Materials and Methods

### Algae Strains

Three marine microalgae strains, two diatoms and a green alga, were chosen to represent different physiologies, biochemistries, and DOC production levels. The diatom strains were *Staurosira* sp. C323 and a novel isolate *Navicula* sp. SFP. Strain C323 was previously used in several studies and classified as *Staurosira* sp. (Ferron et al., 2012; Bittar et al., 2013; Huntley et al., 2015), but we acknowledge new evidence from Li et al. (2018) that may change its taxonomic assignment. The green alga was an isolate identified as *Chlorella* sp. D046. Partial sequences used for identification, in conjunction with microscopy, of *Navicula* sp. SFP and *Chlorella* sp. D046 are deposited in GenBank under accession numbers MK310104 (*Navicula* sp. SFP 18S rRNA) and MK310105, MK317928, MK317845 (*Chlorella* sp. D046 18S rRNA, ITS2, and 23S rRNA, respectively).

### Growth Conditions

Algae were grown in artificial seawater (ASW) modified from Goldman and McCarthy (1978) to have a salinity of 35 ppt, using salts in the following concentrations: 487 mM NaCl, 12.2 mM CaCl_2_, 12.1 mM KCl, 24.3 mM MgCl_2_-6 H_2_O, 24.4 mM MgSO_4_-7 H_2_O, 2.1 mM KBr. Non-hydrated salts were combusted (≥500°C for ≥4 h) before use to reduce potential organic material contamination. Boric acid was added from stock solution to a final concentration of 244 μM H_3_BO_3_. Filter-sterilized (0.2 μm) inorganic nutrient stocks were added to autoclaved ASW in final concentrations of 100 μM Na_2_HPO_4_-H_2_O, 400 μM NH_4_Cl, 4 mM NaHCO_3_, f/2 vitamins, and f/10 trace metals (Andersen et al., 2005). Media for diatoms included 800 μM Na_2_SiO_3_-9 H_2_O. Silicate stock used in media for *Navicula* sp. was acidified to a pH of ~2 for full dissolution (McLachlan, 1973). This acidified stock caused the medium pH to be 6.6, so a new silicate stock was acidified to pH 4.5 for the *Staurosira* sp. experiment, which led to a medium pH of 7.8.

Experimental cultures were grown in 1 L glass media bottles (VWR) in an incubator (Percival) at 25°C with a 12:12 h light:dark cycle at 385 ± 13 μmol photons/m^2^/s. Positions of experimental culture bottles were randomized daily. Culture bottles were plugged with silicone stoppers (Cole-Palmer) equipped with an air inlet, air outlet, and sampling port. Cultures were bubbled with (0.2 μm-filtered) ambient air at 1 vvm for *Chlorella* sp. and *Navicula* sp., and at 0.5 vvm for *Staurosira* sp. due to this alga's sensitivity to aeration.

### Experimental Set-Up

Experiments consisted of five consecutive rounds of batch cultures with a reused medium treatment and a fresh medium control, each grown in triplicate. Batch cultures grew for 5 days in each round. In the first round, both reused medium treatments and fresh medium controls grew in fresh medium. In rounds two through five, reused medium treatments were grown in medium reused from the previous round. Only four rounds were carried out for *Staurosira* sp. due to growth inhibition.

To prepare reused medium, algae were harvested on the final day of each round via vacuum filtration (~15 mm Hg) with 0.45-μm polyethersulfone bottle-top filters (VWR). Filters were pre-rinsed with 500 mL of Nanopure water. Filtrate was collected in sterile bottles and refrigerated at 4°C overnight, then replenished with nutrients. Ammonium, phosphate, and silicate were measured in culture filtrate samples (section Nutrients) to calculate how much to add to reach original concentrations. Trace metals and vitamins were replenished completely. Sodium bicarbonate was added back completely in the *Chlorella* sp. and *Staurosira* sp. experiments, and partially (2 mM) in the *Navicula* sp. experiment.

At the start of each round, both the reused medium treatments and fresh medium controls were inoculated from a single algae culture, which was kept in exponential phase by daily transfers with fresh growth medium (30–50% v/v). *Chlorella* sp. and *Staurosira* sp. experimental cultures were inoculated at 5% v/v, while *Navicula* sp. was inoculated at 7% v/v. Both fresh medium controls and reused medium treatments started with the same volume in each round, which was approximately 1,100, 990, 840, 655, and 470 mL for the first through fifth rounds, respectively.

### Post-experiment Filtrate

At the end of each experiment, all cultures were filtered (0.45 μm) and replenished with nutrients as described in Experimental Set-up. Filtrate was stored in acid-washed 250 mL glass bottles (VWR) in the dark at room temperature (~21°C). Samples were taken periodically for DOC and bacteria concentrations, as described in Sample Collection and Measurement.

### Sample Collection and Measurement

Aliquots were sampled from experimental culture bottles with plastic syringes (BD) under aseptic conditions, and used for the following measurements.

#### Dissolved Organic Carbon (DOC)

Culture aliquots (~25 mL) were sequentially filtered as follows. Approximately 5 mL was filtered through a combusted 25 mm GF/F filter (Whatman) using a 30 mL plastic syringe and a syringe filter holder (PALL #4320). This initial filtrate was disposed of due to DOC adsorption (Moran et al., 1999). The remaining ~20 mL was filtered, and approximately 5 mL of GF/F filtrate was then filtered through a 0.2-μm polycarbonate filter into a 40 mL combusted glass vial. Polycarbonate filters had been pre-soaked in 10% HCl for about 24 h and then in Nanopure water to minimize carbon contamination (Wurl and Sin, 2009). The initial 5 mL of 0.2 μm-filtrate was used to rinse the vial, and then used to measure optical density (OD), salinity, nutrients, and extracellular lipids, as described below. The remaining ~15 mL of GF/F filtrate was filtered through the rinsed polycarbonate filter, collected in the vial, and stored at −4°C.

Samples were measured on a TOC-L analyzer (Shimadzu) with a high-salt combustion kit, using the non-purgeable organic carbon method. Each sample was measured four times with 100 μL injection volumes. A reference solution (TOCKHP1, Inorganic Ventures) was used to prepare DOC standards. Total dissolved nitrogen was also measured simultaneously with a TNM-L module, using KNO_3_ solution as a standard [data not shown, but available in corresponding data repository (Loftus and Johnson, 2018)]. DOC in ASW media (average 38.6 ± 7.6 μM, *n* = 32 batches) was subtracted from samples to calculate the biologically-derived DOC concentration. DOC in nutrient additions was also calculated in each experiment (average 26.1 ± 7.4 μM, *n* = 22 batches) and subtracted from reused medium treatments.

#### Neutral Lipids

Culture aliquots and 0.2-μm filtrate were measured for total lipids and extracellular lipids, respectively, following Johnson et al. (2017). Briefly, samples were preserved with 5 μL/mL of 25% glutaraldehyde (final concentration 0.12%) and stored at −80°C. Relative neutral lipid concentrations were measured by staining with Nile Red (9-diethylamino-5H-benzo-α-phenoxazine-5-one) (Sigma-Aldrich) and measuring fluorescence. A stock solution of 1 mg Nile Red per 1 mL DMSO was added to samples in a final concentration of 6 μg Nile Red per mL, and samples were measured using a Synergy 4 plate reader (Biotek). Three technical replicates were measured and averaged for each sample.

#### Dissolved Inorganic Carbon

Culture aliquots were stored in combusted 5 mL amber vials, to which saturated mercuric chloride solution was added at 0.04% v/v (Dickson et al., 2007). Samples were measured as in Johnson et al. (2013) with an Apollo Scitech DIC analyzer (model AS-C3) with an attached Li-Cor 7000 CO_2_ detector, using 6% v/v phosphoric acid with 1.71M NaCl to acidify samples. Certified Reference Materials purchased from Dr. A. G. Dickson (Scripps Institution of Oceanography, University of California) were used to make standard curves. Each DIC sample was measured 3–4 times by 1 mL injections, accepting only the last measured injection. Replicate samples were taken for each *Navicula* sp. culture and, after replicate variability proved to be low (typically <1% CV), single samples were taken per *Staurosira* sp. culture.

#### Carbon Production Rates

Culture aliquots (4.5 mL) were transferred to dark plastic bottles to measure light-driven carbon production rates with radiolabelled carbon, a method originating from Steeman Nielsen (1952) and following Johnson and Sheldon (2007). Approximately 92.5 kBq NaH^14^CO_3_ was added to each sample, which was mixed and transferred to four borosilicate glass vials (1 mL per vial). Vials were incubated under culturing conditions, with two vials per sample kept dark. After a 2-h incubation, half of each vial volume was vacuum-filtered through a pre-wetted glass fiber filter (~1.6 μm, VWR 691). Filters were rinsed with ASW and placed in clean glass vials to determine particulate carbon production. Remaining sample volume was kept to determine total carbon production. All vials were stored uncapped in the dark overnight after addition of 50 μL 37% formaldehyde and 100 μL of 1N HCl. Replicate 10 μL samples of NaH^14^CO_3_ stock solution were taken to measure the stock's exact radioactivity, after adding two drops of beta-phenylethylamine and 3 mL scintillation cocktail (Ecolume, MP Biomedicals #01882470).

After overnight storage, 3 mL of scintillation cocktail was added to each sample vial. Vials were measured on a Beckman Coulter LS 6500 scintillation counter for 5 min each. Average disintegrations per minute (DPM) of dark-incubated replicates were subtracted from average DPM of corresponding light-incubated replicates to calculate sample DPM. Production rates were calculated as in Johnson and Sheldon (2007), except using measured values of DIC.

DOC release rate was calculated by subtracting the particulate organic carbon production rate (from filter samples) from the total organic carbon (TOC) production rate (from whole culture samples). Relative carbon production rates for *Chlorella* sp. are only available for the final round of the experiment due to a defective ^14^C stock, and absolute rates are unavailable because of lack of DIC data.

#### Photosynthetic Efficiency

Culture aliquots in 50 mL glass tubes were kept dark for ~20 min. Tubes were then placed in a dark casing connected to a FIRe (fluorescence induction and relaxation) fluorometer equipped with a fiber optic probe (Satlantic). The FIRe system was used to evaluate photosynthetic physiology via an initial 200 μs pulse from a blue LED excitation source. ASW growth medium was measured as a blank. Data from the FIRe fluorometer were processed with a customized MATLAB script based on Kolber et al. (1998) and Johnson (2004) to estimate the maximum quantum yield of photochemistry in Photosystem II, or F_v_/F_m_, from a fluorescence induction model.

#### Particulate Carbon

GF/F filters from initial filtering of DOC samples were stored at −80°C in mini petri plates lined with combusted aluminum foil. Filters were dried for at least 24 h at 60°C and pelletized in 4 × 10 mm tin capsules (Costech). Pellets were sent to the Duke Environmental Stable Isotope Laboratory where they were analyzed on a CE FlashEA 1112 Nitrogen and Carbon Analyzer (Thermo Finnigan). Carbon and nitrogen masses measured on blank filters, which were filtered with growth medium, were subtracted from samples. C and N masses were divided by filtered sample volume to calculate C and N concentrations. Particulate N data are not reported here but are available in the corresponding data repository (Loftus and Johnson, 2018).

#### Bacteria and Algae Cell Concentrations

Culture aliquots were preserved with 5 μL/mL of 25% glutaraldehyde (final concentration 0.12%) and stored at −80°C (Marie et al., 1999). Algae and bacteria cell concentrations were measured with a FACSCalibur flow cytometer (Becton Dickson), as described in Johnson et al. (2010). Samples were injected at a flow rate of 10 μL/min (or 50 μL/min for *Staurosira* sp. and *Navicula* sp. algae samples) from a 1 mL plastic syringe (Becton Dickson). Bacteria samples were stained with SYBR Green I stain (final concentration 2.2x) for at least 30 min (modified from Marie et al., 1999).

Cells were identified based on fluorescence and side scatter using FlowJo software version 7.6.5. Cell concentrations were calculated as in Marie et al. (1999), with the correction that cell count was divided by sample run time. Average bacteria concentrations in SYBR-stained growth medium samples were subtracted from those measured in culture samples to account for sample carryover and noise.

#### Nutrients

Culture filtrate (0.2 μm) samples were stored frozen at −80°C until measurement. Phosphate and silicate concentrations were measured on the plate reader following previously described methods (Ringuet et al., 2011), with the modification that silicate reactions took place in 1.7 mL centrifuge tubes and were centrifuged at 10,000 *g* for 5 min prior to transferring to a 96-well plate. Ammonium concentrations were measured using the fluorometric method of Holmes et al. (1999) adapted for the plate reader. One hundred microliter of sample or standard was added to 100 μL of reagent in wells of a black 96-well plate, and the plate was shaken at 450 rpm on a plate shaker (MTS 2/4 digital, IKA) for at least 3 h.

For each method, samples and standards were measured in triplicate wells. Standard working solutions were prepared in ASW. Samples were diluted with ASW if they exceeded maximum measurable concentrations, which were ~50 μM PO_4_, ~50 μM NH_4_, and ~100 μM Si.

#### Optical Density (OD)

Whole culture and culture filtrate (0.2 μm) OD was measured at 750 nm in clear, flat-bottom 96-well plates (Corning #3596) using the plate reader, with triplicate wells per sample (200 μL/well). ASW growth medium was measured as a blank.

#### Salinity and pH

Salinity of culture filtrate (0.2 μm) was measured on a pocket refractometer (Atago PAL-06S). pH of whole culture aliquots was measured with a calibrated probe (VWR sympHony).

#### Bacteria Carbon Consumption

Carbon consumption by bacteria was estimated from the change in bacteria cell concentration over each round. We assumed an average bacteria carbon content of 100 fg C/cell [a low estimate based on laboratory cultures in Bratbak (1985) and Vrede et al. (2002)] and a bacteria growth efficiency of 0.15 [an estimate based on references reviewed by del Giorgio and Cole (1998) and Rivkin and Legendre (2001)].

### Statistical Analyses

Analyses were performed in RStudio version 1.1.383 using R version 3.3.3 (R Core Team, 2017) and made use of the tidyverse package (Wickham, 2017). Data are publicly available in a Figshare repository (Loftus and Johnson, 2018) and code is publicly available on GitHub (Loftus, 2018). In statistical tests, a *p*-value below 0.05 was considered significant.

#### Algae Biomass Production in Reused Medium

Four variables relevant to industrial algae biomass production were assessed: (1) biomass yield, calculated as the change in particulate carbon from Day 0 to 5 in each round (μM C); (2) specific growth rate, calculated as the slope of the natural log of cell concentration vs. time, during exponential phase only (Days 0–2 for *Chlorella* sp. and Days 0–3 for *Navicula* sp. and *Staurosira* sp.) (day^−1^); (3) lipid content, calculated as the biomass lipid fluorescence divided by algae cell concentration on Day 5 of each round (RFU/(cells/mL)); and (4) photosynthetic efficiency, F_v_/F_m_, on Day 5 of each round (unitless).

To determine whether medium reuse affected these variables, and whether the number of reuses influenced this effect, a linear mixed effects model was defined for each response variable using the nlme package in R (Pinheiro et al., 2017). Predictor variables were the experimental treatment (fresh vs. reused medium) and round of the experiment (excluding the first round in which both the control and treatment were grown in fresh medium). Random effects were the replicate bottles, and an autocorrelation structure of corAR1 was used with culture round as the time variable and replicate bottles as the subjects, to account for dependency of reused media across rounds (although fresh medium controls were independent across rounds). A Type II Wald test was performed to determine significance of the predictor variables using the “Anova” function in the car package (Fox and Weisberg, 2011; Mangiafico, 2016).

Particulate carbon and lipid data were unavailable for some replicates on Day 5 of a culture round. These included particulate carbon data from all replicates of *Navicula* sp.'s fresh medium control in Round 4, lipid data from one replicate of *Navicula* sp.'s reused medium treatment in Round 2, and lipid data from one replicate of *Staurosira* sp.'s fresh medium control in Round 1. In these cases, missing data were estimated based on linear regression equations of algae cell concentration with the missing variable, calculated using all other sample data from the experiment (or in the case of lipids, only data from Day 5 of each round).

#### Effect of Medium Reuse on Algal DOC Release

To determine whether medium reuse affected algal DOC release, and if number of reuses influenced this effect, a linear mixed effects model was defined as in Algae Biomass Production in Reused Medium. The response variable was DOC released within a round, normalized by TOC produced. Released DOC and produced TOC were both predicted by multiplying measured DOC release rates and TOC production rates, respectively, by the light period (assuming negligible release and production in the dark). On days where rates were not measured (Days 1 and 3 of each round), an average of rates from the preceding and following day was used. Here, released DOC refers to the gross amount of DOC released by algae, which is distinguished from accumulated DOC, defined as the net amount of DOC in the growth medium.

#### Relationship Between DOC Concentration and Algae Growth in Reused Medium

To determine if algae biomass yield in reused medium was correlated with biologically-derived DOC concentration, another linear mixed effects model was defined using the nlme package in R (Pinheiro et al., 2017). The response variable was biomass yield (described in Algae Biomass Production in Reused Medium) in reused medium treatments normalized by mean biomass yield in fresh medium treatments from the same round. The predictor variable was the initial biologically-derived DOC concentration in reused medium treatments. The first round of the experiment was included in which the initial biologically-derived DOC was solely from the inoculum. Random effects were the replicate bottles nested within algae taxa. An autocorrelation structure of corAR1 was used with round as the time variable and the subjects defined as replicate bottles nested within algae taxa. A Type II test was performed as described in Algae Biomass Production in Reused Medium.

## Results

### Algal Biomass Production in Reused Medium

**Figure 1:**
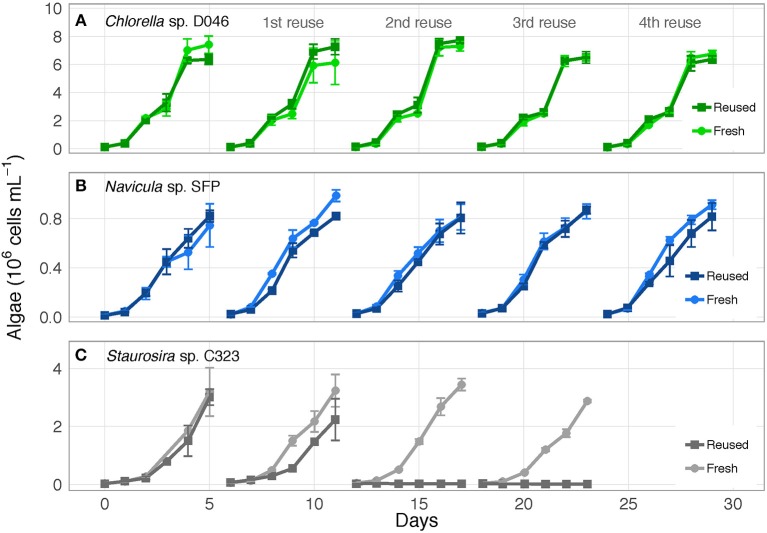
Algae cell concentrations over time for **(A)** *Chlorella* sp. D046, **(B)** *Navicula* sp. SFP, and **(C)** *Staurosira* sp. C323 in fresh and reused growth medium. Error bars represent standard deviation of three replicate cultures. Data from one to three of the replicates of *Staurosira* sp. C323 are unavailable from Reused and Fresh treatments on Days 3 and 4.

The three algae exhibited different responses to reused growth medium. *Chlorella* sp. and *Navicula* sp. showed no major changes in cell concentrations between fresh and reused medium, while *Staurosira* sp. experienced severe growth inhibition by the second reuse of the medium (Figure 1; OD_750_ results in Figure S1). Additionally, while *Chlorella* sp. and *Navicula* sp. reused medium treatments supported higher bacteria concentrations than fresh medium cultures, bacteria in *Staurosira* sp. reused medium reached lower concentrations than in fresh medium by the third reuse (Figure S2).

**Figure 2:**
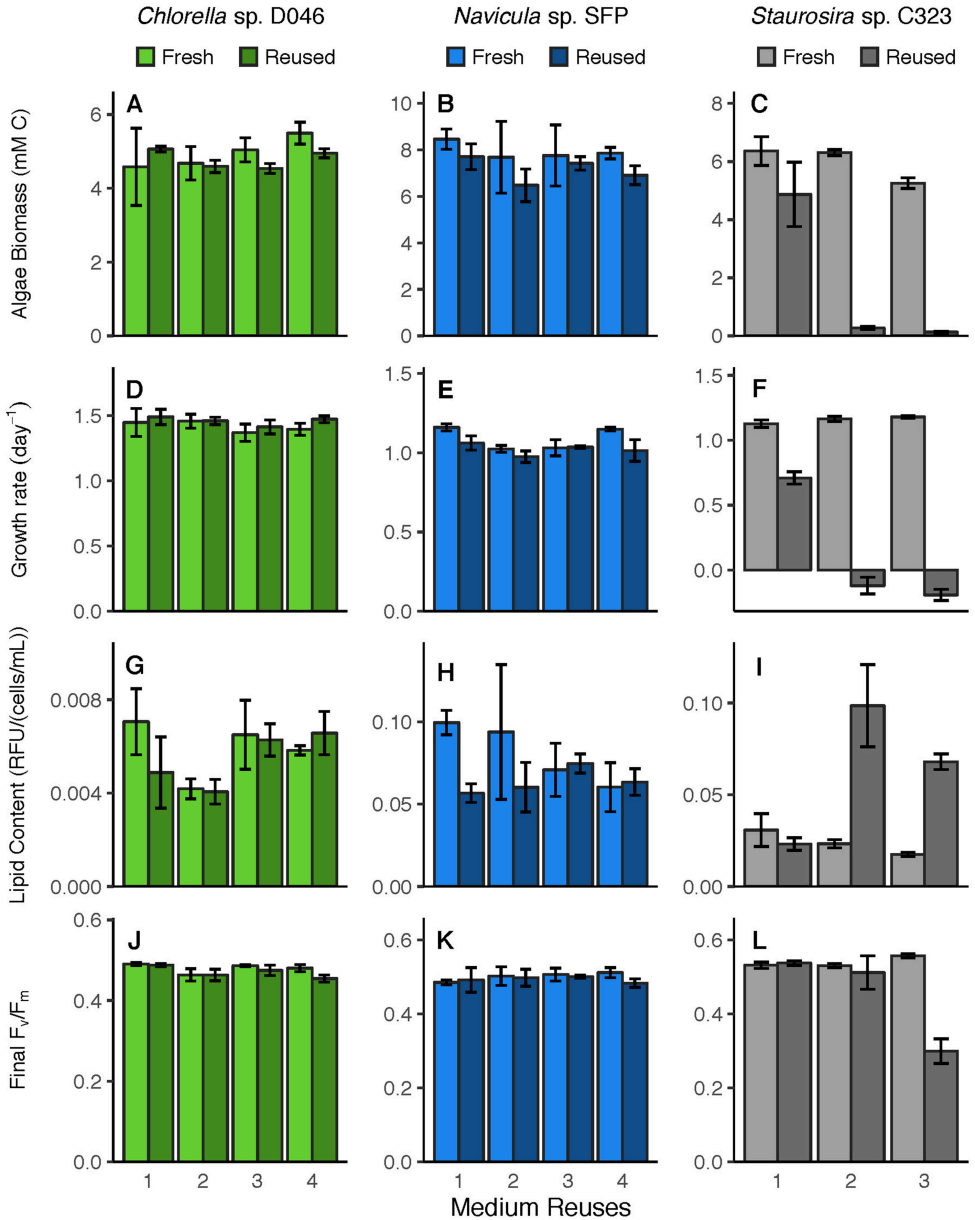
Parameters relevant to industrial algae production, including **(A–C)** biomass yield (mM C), **(D–F)** specific growth rate (day^−1^), **(G–I)** lipid content (RFU/(cells/mL)), and **(J–L)** photosynthetic efficiency, or F_v_/F_m_, in fresh medium controls and reused medium treatments. Error bars represent standard deviation of three replicate cultures.

Algal response variables (biomass yield, specific growth rate, lipid content, photosynthetic efficiency) (Figure 2) were examined for overall differences between fresh and reused medium, and for interaction effects between treatment and number of medium reuses. None of these parameters were significantly different between fresh and reused treatments overall for *Chlorella* sp. Parameters were also not different for *Navicula* sp. except for specific growth rate [$\chi_{\left(1\right)}^{2}$ = 10.62, *p* = 0.001]. Differences between *Navicula* sp. growth rates in fresh vs. reused medium were not consistent across rounds however, meaning the significance was likely not driven by sustained toxicity of reused medium. For *Staurosira* sp., all parameters were significantly different overall in reused medium, including biomass yield [$\chi_{\left(1\right)}^{2}$ = 518.4, *p* < 0.0001], specific growth rate [$\chi_{\left(1\right)}^{2}$ = 4022.6, *p* < 0.0001], final lipid content [$\chi_{\left(1\right)}^{2}$ = 46.55, *p* < 0.0001], and final F_v_/F_m_ [$\chi_{\left(1\right)}^{2}$ = 26.06, *p* < 0.0001].

Additionally, all parameters for *Staurosira* sp. exhibited a significant interaction effect between treatment and medium reuses, meaning the reused medium had a differential effect based on how many times it was reused. These interaction results included biomass yield [$\chi_{\left(1\right)}^{2}$ = 27.16, *p* < 0.0001], specific growth rate [$\chi_{\left(1\right)}^{2}$ = 1009.9, *p* < 0.0001], final lipid content [$\chi_{\left(1\right)}^{2}$ = 109.1, *p* < 0.0001], and final F_v_/F_m_ [$\chi_{\left(1\right)}^{2}$ = 159.4, *p* < 0.0001]. Some parameters for *Navicula* sp. and *Chlorella* sp. also had significant interaction effects though the parameters were not significantly different in fresh vs. reused medium, meaning that these effects are simply from variation across rounds. *Navicula* sp. had a significant interaction effect for final lipid content [$\chi_{\left(1\right)}^{2}$ = 26.89, *p* < 0.0001] only, and *Chlorella* sp. had significant interaction effects for biomass yield [$\chi_{\left(1\right)}^{2}$ = 4.61, *p* = 0.032], final lipid content [$\chi_{\left(1\right)}^{2}$ = 10.42, *p* = 0.001], and final F_v_/F_m_ [$\chi_{\left(1\right)}^{2}$ = 9.23, *p* = 0.002].

Environmental conditions, including pH (Figure S3) and DIC (Figure S4), were not identical in reused and fresh medium treatments, although these likely did not cause growth inhibition of *Staurosira* sp. Salinity (Figure S5) and OD (Figure S6) of the media did not increase with reuse.

### Effect of Medium Reuse on DOC Release and Accumulation

**Figure 3:**
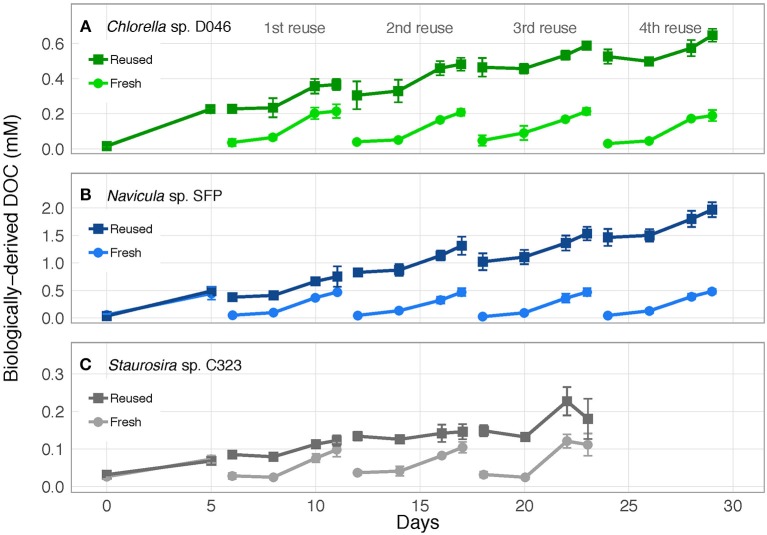
Concentration of accumulated, biologically-derived DOC in cultures of **(A)** *Chlorella* sp. D046, **(B)** *Navicula* sp. SFP, and **(C)** *Staurosira* sp. C323 in fresh and reused growth medium. Error bars represent standard deviation of three replicate cultures.

**Figure 4:**
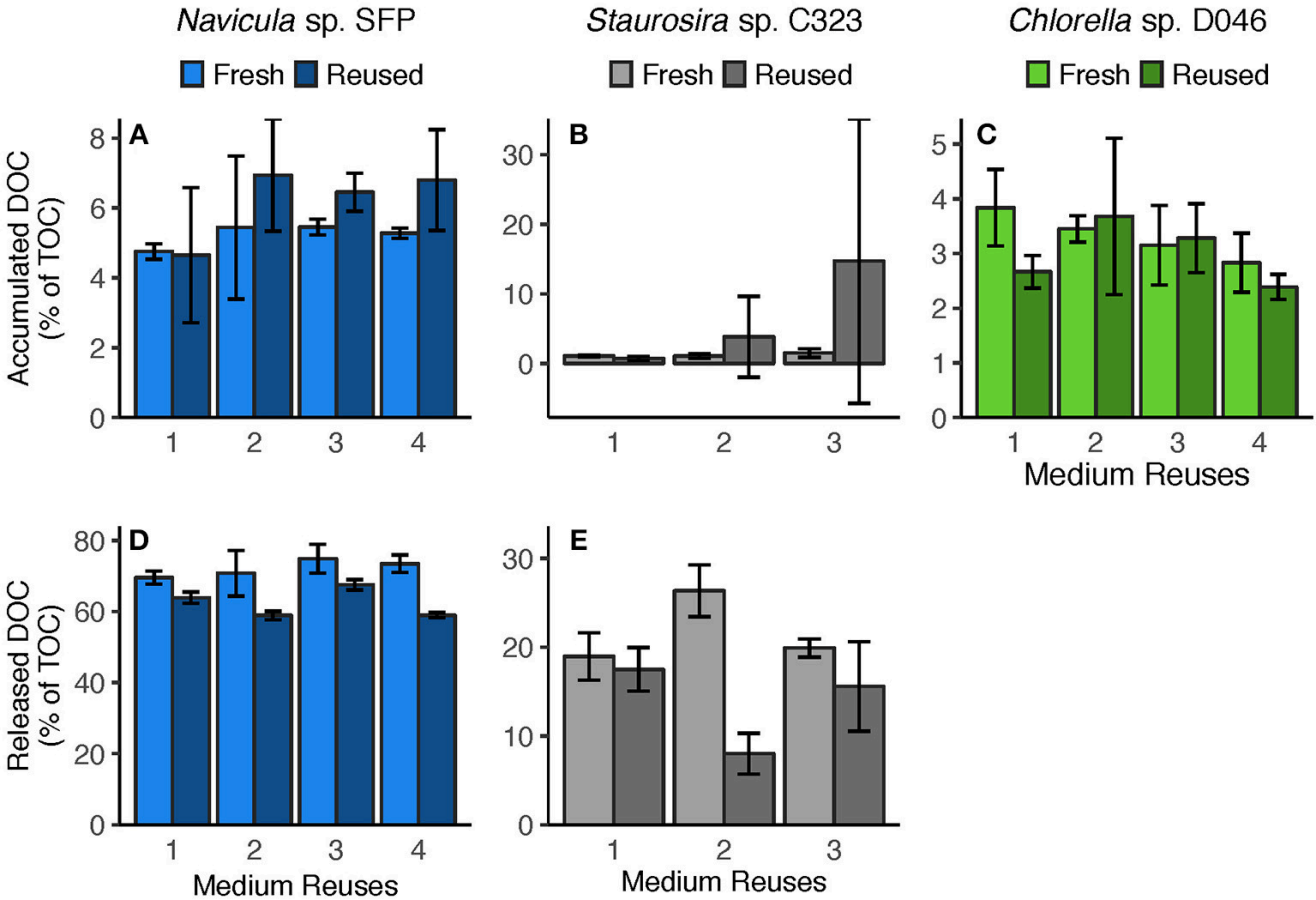
**(A–C)** Accumulated DOC (difference between Day 0 and Day 5) and **(D,E)** released DOC (predicted from DOC release rates) per round. Accumulated DOC was normalized by the sum of accumulated DOC and particulate carbon yield. Released DOC was normalized by TOC produced (estimated from TOC production rates). Error bars represent standard deviation of three replicate cultures. Note: *Chlorella* sp.'s Reused Treatment in Reuse 2 is from 2 replicates only. Released DOC data are unavailable for *Chlorella* sp.

Biologically-derived DOC accumulated consistently with each medium reuse, except when *Staurosira* sp. didn't grow in Rounds 3 and 4 (Figure 3). Net DOC accumulation showed no trends across reuses for *Navicula* sp. and *Chlorella* sp. (Figures 4A,C). The variability in DOC accumulation increased across reuses as *Staurosira* sp. became growth inhibited (Figure 4B).

Medium reuse had a significant overall effect on *Navicula* sp.'s predicted gross DOC release, normalized by TOC produced [$\chi_{\left(1\right)}^{2}$ = 31.02, *p* < 0.0001] (Figure 4D). There was no significant interaction effect between treatment and round [$\chi_{\left(1\right)}^{2}$ = 3.478, *p* = 0.062], however, indicating that the effect of reused medium did not intensify over multiple reuses. *Staurosira* sp. also had significantly different released DOC concentrations in reused medium when normalized by TOC produced [$\chi_{\left(1\right)}^{2}$ = 194.7, *p* < 0.0001], and no significant interaction between treatment and culture round [$\chi_{\left(1\right)}^{2}$ = 1.682, *p* = 0.195] (Figure 4E). Comparing concentrations of accumulated DOC and predicted released DOC (Figure S9), accumulated DOC accounted for <10% of released DOC for *Navicula* sp. and Round 2 *Staurosira* sp. cultures, indicating loss of DOC (see DOC Degradation in Reused Medium in Discussion).

Average gross DOC release rates for *Navicula* sp. ranged from 0.07 to 5.4 mM C/day (Figure S7A) and 32 to 85% of the TOC production rate (Figure S8B) across all treatments, peaking in early exponential phase. For *Staurosira* sp., average DOC release rates ranged from 0.001 to 1.4 mM C/day (Figure S7B) and 5 to 51% of the TOC production rate (Figure S8C), with a peak on Days 4 or 5 of the round. *Chlorella* sp.'s DOC release rates in the final round ranged from 8 to 25% of the TOC production rate, peaking on Day 4 (Figure S8A).

### Relationship Between DOC Concentration and Algae Yield in Reused Medium

To determine whether there was a broad relationship between accumulated DOC concentration in reused medium and the medium's suitability for algae cultivation, we collectively analyzed biomass yields from the three algae strains. Biomass yields in reused medium were normalized by the mean biomass yield in fresh medium from the same round, so that values would be comparable across algae. There was no significant relationship between biomass yield and initial DOC concentration [$\chi_{\left(1\right)}^{2}$ = 1.0918, *p* = 0.2961] (Figure S10), even after removing *Staurosira* sp.'s third and fourth round in which algae failed to grow [$\chi_{\left(1\right)}^{2}$ = 2.96, *p* = 0.086]. Indeed, *Staurosira* sp. had the lowest biologically-derived DOC concentrations in reused medium yet the poorest growth.

### DOC Degradation in Reused Medium

Net DOC accumulation in each round represented <10% of predicted gross DOC released by *Navicula* sp. and *Staurosira* sp. cultures in which algae grew, meaning a >90% loss of released DOC. Estimated bacteria carbon consumption (Bacteria Carbon Consumption) accounts for a portion of the lost DOC. In *Navicula* sp. reused medium cultures, bacteria carbon consumption ranged from only 20 ± 3 to 31 ± 2% of the lost DOC, depending on the round. In the reused medium treatment of *Staurosira* sp. from Round 2, bacteria consumption represented 75 ± 15% of lost DOC.

**Figure 5:**
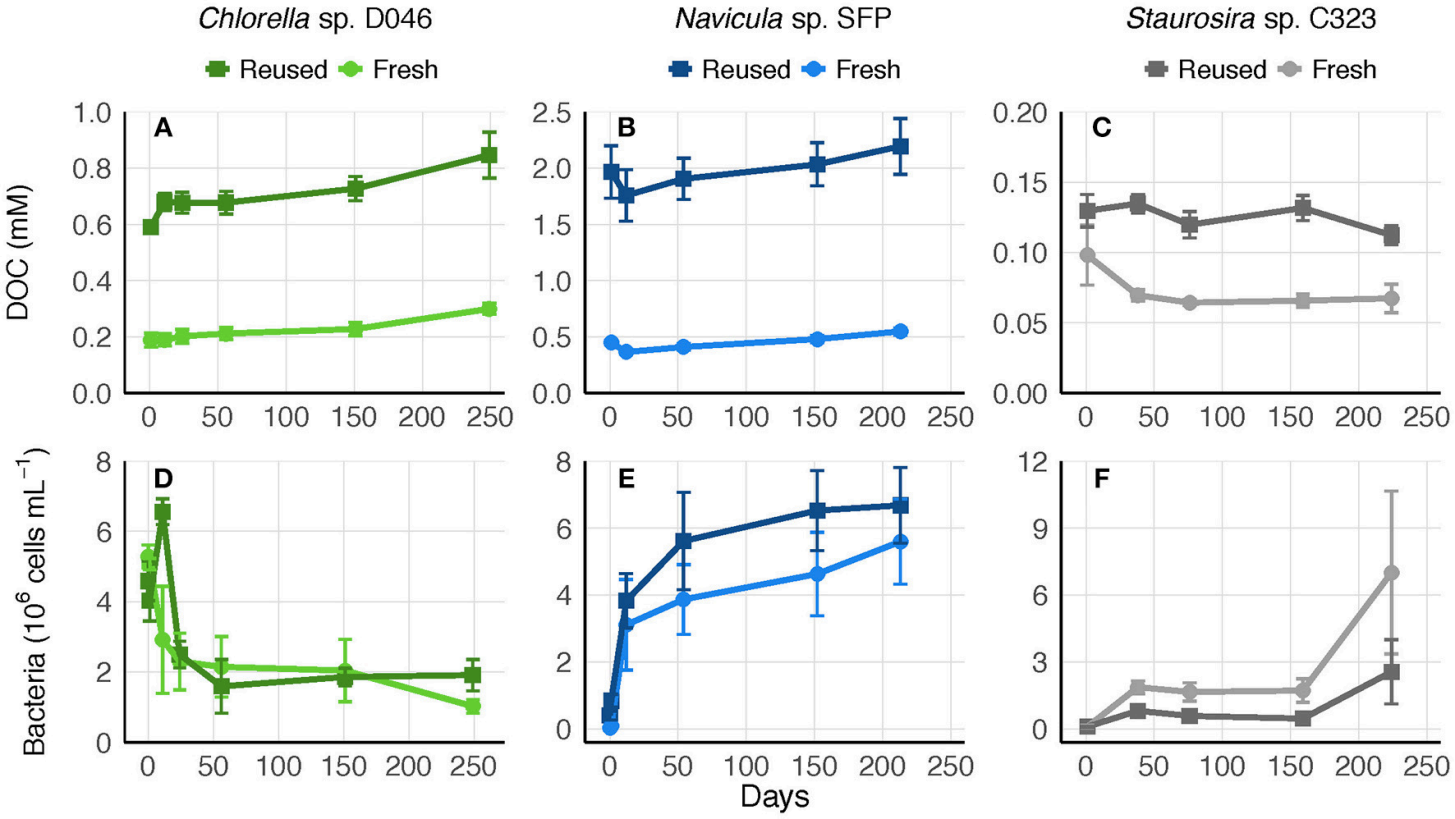
**(A–C)** Biologically-derived DOC concentration and **(D–F)** bacteria concentration in 0.45-μm filtrate of fresh and reused growth medium cultures saved after the final round of each experiment. Error bars represent standard deviation of three replicate cultures.

To determine whether the accumulated DOC was bioavailable or recalcitrant to further degradation, 0.45-μm culture filtrate was saved at the end of experiments and replenished with nutrients. Bacteria remaining in the 0.45-μm filtrate represented an average of 39, 1, and 1% of bacteria concentrations from the final experimental cultures of *Chlorella* sp., *Navicula* sp., and *Staurosira* sp., respectively. DOC concentrations in the filtrate remained relatively stable for over 200 days, indicating no net transformation to carbon dioxide. Bacteria concentrations increased or remained stable over the majority of the time period in both fresh and reused medium treatments, though *Chlorella* sp. bacteria decreased within the first 50 days (Figure 5).

## Discussion

### Algal Biomass Production in Reused Medium

Algae growth responses observed here are broadly consistent with the observation that growth success in reused medium is taxa-dependent, as shown in a meta-analysis (Loftus and Johnson, 2017). This genus-level specificity appears to be true even within major groups of algae, as within the diatoms *Staurosira* sp. showed severe growth inhibition but *Navicula* sp. did not.

*Staurosira* sp.'s neutral lipid content was also higher in the second and third medium reuse, a potential sign of stress caused by unfavorable conditions during which algae divert carbon to storage molecules (Sharma et al., 2012; Figure 2I). Since initial nutrient concentrations were equivalent in fresh and reused medium, the cause of stress may have been toxicity of accumulated DOC from algae and/or bacteria. Lysed algae cells could have released harmful DOC, although extracellular lipids, which could be a sign of ruptured cells, did not increase considerably across reuses [data not shown, but available in corresponding data repository (Loftus and Johnson, 2018)].

Growth inhibition of *Staurosira* sp. could also be driven by bacteria transforming DOC or cell debris into other inhibitory compounds. Inhibited *Staurosira* sp. eventually grew when inoculated back into fresh medium (data not shown), ruling out a bacteria or viral infection that completely killed the algae. Future studies will test the strain-specificity of the inhibitory mechanism of *Staurosira* sp. reused medium, and determine how the alga's microbiome differs from that of the other tested algae.

Bacteria still grew in *Staurosira* sp. reused medium even when algae could not, although by the third medium reuse bacteria reached a 45% lower mean final concentration than in the control (Figure S2), which could indicate the reused medium was toxic to some or all of the coexisting bacteria strains. Alternatively, a lack of labile DOC output from *Staurosira* sp. could have also caused the relatively low bacteria concentrations. In all other reused medium cultures across the three algae, bacteria reached 33% higher final concentrations than in fresh medium on average, likely due to higher DOC concentrations and residual bacteria in the reused medium.

### Effect of Medium Reuse on DOC Release and Accumulation

Understanding DOC production dynamics in reused medium is important because certain types of DOC can affect algae growth (e.g., Burkiewicz and Synak, 1996; Zhang et al., 2013, 2016). The consistent DOC accumulation observed under conditions tested here suggests a build-up of recalcitrant DOC across sequential medium reuses (Figure 3).

We measured algal DOC release rates to determine if they are affected by reused medium, possibly because of a feedback effect from accumulated DOC in the water. During early exponential phase, *Navicula* sp.'s average DOC release rate was lower in the second, third, and fourth medium reuse compared to that in fresh medium (Figures S7A, S8B). These differences caused *Navicula* sp.'s predicted gross released DOC concentration, normalized by TOC, to be significantly different overall in fresh vs. reused medium (Figure 4D). Gross DOC release predictions are based on interpolation of DOC release rates throughout the culture round. Nevertheless, this change in DOC release could signify physiological and/or metabolic changes caused by medium reuse, and/or a decreased concentration gradient that slowed passive diffusion out of cells. DOC release rates did not decrease proportionally to the increase in accumulated DOC, however, so there was no concentration-dependent feedback effect, but potentially a threshold effect.

Similarly, *Staurosira* sp. exhibited a significant difference in gross released DOC, normalized by TOC, between fresh and reused medium overall, though there was also no trend of decreasing DOC release across reuses (Figure 4E). Although carbon production rates are unavailable for *Chlorella* sp., the fraction of fixed carbon released as DOC was similar in fresh and reused medium in the final round of the experiment (Figure S8A), suggesting no effects on DOC release rates. It may also be plausible that reused media did not reach high enough DOC concentrations for concentration-dependent feedback effects to occur among these algae. Nevertheless, lack of a concentration-dependent feedback on DOC release means that DOC production and accumulation would not slow as growth medium is continually reused.

DOC release rates observed for *Navicula* sp. represented a large fraction of TOC production (about 40 to 80%), while *Staurosira* sp.'s relative DOC release rates (about 5 to 50% of TOC production) were more comparable to values reported in the literature, which range from 7 to 37% (Obernosterer and Herndl, 1995; Biddanda and Benner, 1997; Hulatt and Thomas, 2010). *Navicula* sp.'s relatively high percent DOC release rate could have several explanations. *Navicula* is known for biofouling and secretes adhesive compounds made of carbohydrates and proteins (Staats et al., 1999) to attach to surfaces and glide (Molino and Wetherbee, 2008). We observed that *Navicula* sp. created a durable biofilm on glass bottles when not agitated by aeration or mixing. It is plausible that *Navicula* sp. has an exceptionally high percent DOC release compared to other algae, especially in the agitation-free environment during ^14^C incubation in vials. Additionally, we've observed mixotrophic capabilities in *Navicula* sp. as it grew on agar plates (with Difco AC broth) in the dark, so it is possible that the alga releases DOC during peak growth to consume later, as has been shown in cyanobacteria mats (Stuart et al., 2016). Lastly, although care was taken to prevent cell breakage during ^14^C sample filtering, it is also possible that some *Navicula* sp. cells ruptured and their radiolabelled contents were not retained on the filters, thereby underestimating particulate carbon production rates and overestimating DOC release rates.

### Relationship Between DOC Concentration and Algae Yield in Reused Medium

Other studies have found associations between DOC concentration and algae growth (Zhang et al., 2013, 2016; Depraetere et al., 2015; Christie-Oleza et al., 2017). However, these studies each used a single algae strain. Here we found that DOC concentration is not correlated with algae growth response across physiologically different algae taxa, suggesting the quality of reused media likely cannot be predicted from DOC concentration alone.

Final DOC concentrations in reused medium cultures were somewhat low (0.2, 0.65, and 2 mM for *Staurosira* sp., *Chlorella* sp., and *Navicula* sp., respectively) compared to those measured in other studies, which ranged from about 0.4 to 8 mM (Hulatt and Thomas, 2010; Depraetere et al., 2015; Wu et al., 2016; Zhang et al., 2016). Lower DOC concentrations could be due to lower nutrient concentrations and/or shorter growth periods used in our experiments, in addition to different algal taxonomies. Given the variety of growth responses observed in studies that measured higher DOC concentrations [e.g., neutral and stimulatory responses for *Ankistrodesmus* (Hesse et al., 2017), and inhibitory responses for *Arthrospira* (Depraetere et al., 2015) and *Scenedesmus* (Zhang et al., 2013)], the lack of a significant relationship between growth response and DOC concentration should not change if these studies were included. If DOC concentration is not broadly predictive of reused medium suitability, other factors could be more influential such as the molecular composition of DOC, identity of coexisting bacteria, or accumulation of micronutrients to toxic concentrations.

### DOC Degradation in Reused Medium

The degree to which material in reused medium is recalcitrant or labile will affect DOC accumulation and bacteria growth. Less than 10% of predicted DOC released by *Staurosira* sp. and *Navicula* sp. accumulated in reused media in each round. Estimated consumption of DOC by bacteria accounted for a portion of the lost DOC (section DOC Degradation in Reused Medium in Results), but not all, though these consumption estimates are sensitive to values of bacteria carbon content and growth efficiency. The unaccounted for DOC could be explained by several factors. First, measured bacteria concentrations could underestimate true concentrations, since flow cytometer measurements do not include algae- and debris-attached bacteria. Accounting for attached bacteria would increase the amount of DOC consumed. Second, the diatoms could also be consuming DOC, as *Navicula* sp. can grow in the dark (section Effect of Medium Reuse on DOC Release and Accumulation in Discussion). Lastly, interpolating carbon production rates for Days 1 and 3 could have overestimated released DOC.

A biodegradation experiment was performed with post-experiment filtrate to determine what proportion of the remaining DOC was recalcitrant. DOC concentrations did not decrease substantially over the course of >200 days in either reused or fresh medium treatments, but in all cases bacteria concentration either increased or remained stable over the majority of time (Figure 5). Previous biodegradation experiments conducted with environmental samples defined DOC as recalcitrant if it remained after 112 days (Wear et al., 2015) or 150 days (Kragh and Sondergaard, 2009). However, bacteria break down DOC and cell debris in a cycle of uptake and remineralization (Azam et al., 1994), and release DOC as well (Carlson and Hansell, 2015). The lack of net change in DOC observed here may have been caused by bacteria recycling limited bioavailable DOC, releasing their own DOC through excretion or cell lysis, and/or preferentially using labile carbon sources that were 0.2–0.45 μm and therefore not measured as DOC (such as dead bacteria or algal debris that passed through the 0.45-μm filters). Initial bacteria concentrations in the filtrate were also considerably lower than in algae cultures (section DOC Degradation in Reused Medium in Results), so filtration could have selectively removed a large proportion of bacteria able to consume the accumulated DOC. Even so, the lack of net change suggests that the DOC pool was mostly recalcitrant to bacteria in the filtrate. The stability of DOC in culture filtrates observed here suggests accumulated DOC will increase linearly with medium reuse, as was observed in experiments.

## Conclusions

The range of algal growth responses observed here in reused medium confirms taxa-specificity and suggests that algae screening processes for large-scale cultivation should include reused medium testing. This is the first study to analyze the relationship between growth response and biologically-derived DOC concentration in reused medium across physiologically different algae. DOC concentration alone did not predict growth responses, which are likely influenced by more complex factors such as the molecular composition of DOC or interactions with other microbes. This is also the first study to show how reused medium affects algal DOC release rates in comparison to DOC accumulation. The diatom *Navicula* sp. released less DOC in reused medium overall, though there was no interaction effect with the number of medium reuses. Across all three algae, DOC accumulated consistently with each medium reuse, and the remaining DOC was not biodegraded within 200 days after experiments ended, suggesting that it was recalcitrant. Algae screening processes may also need to measure algal DOC release dynamics to choose algae strains and harvesting schedules that minimize DOC released into the medium and maximize carbon harvested in algal biomass. In cases of industrially-advantageous algae that create self-inhibitory reused medium, further strategies could be tested such as algal crop rotation (alternating different algae strains in the same reused medium), harvesting by membrane filtration (Bhujade et al., 2017), or pretreating the water such as with activated carbon (Zhang et al., 2016) or advanced oxidation processes (Wang et al., 2018). Overall, if algae strains do not exhibit inhibition in reused medium screening processes, they can likely be grown reliably in continually reused medium. Medium reuse would allow the algae product industry (fuels, feed, and food) to reduce costs associated with pumping water, treating wastewater, and adding nutrients, and reduce the water footprint of algae cultivation to improve environmental sustainability and allow for growth in more water-limited regions.

## Data Availability

The datasets generated for this study can be found on FigShare along with meta-data at https://doi.org/10.6084/m9.figshare.7237553.v1. The code generated to analyze the data can be found at http://doi.org/10.5281/zenodo.2253460. Algae DNA sequences can be found in GenBank under accession numbers MK310104, MK310105, MK317845, MK317928.

## Author Contributions

SL and ZJ conceived the idea of the study. SL conducted experiments, analyzed data, and wrote the paper. SL and ZJ edited the paper.

### Conflict of Interest Statement

The authors declare that the research was conducted in the absence of any commercial or financial relationships that could be construed as a potential conflict of interest.
