# Supplementary material for: Reused Cultivation Water Accumulates Dissolved Organic Carbon and Uniquely Influences Different Marine Microalgae
Source: Front Bioeng Biotechnol. 2019 May 14;7:101. doi: 10.3389/fbioe.2019.00101 (PMC6528441; doi:10.3389/fbioe.2019.00101)
Supplement: Supplementary file 1 [file Data_Sheet_1.pdf]

Supplementary Material

Reused Cultivation Water Accumulates Dissolved Organic Carbon And Uniquely Influences Different Marine Microalgae

Sarah E. Loftus*, Zackary I. Johnson

*** Correspondence:** Sarah E. Loftus: sarah.e.loftus@gmail.com

1 Supplementary Figures

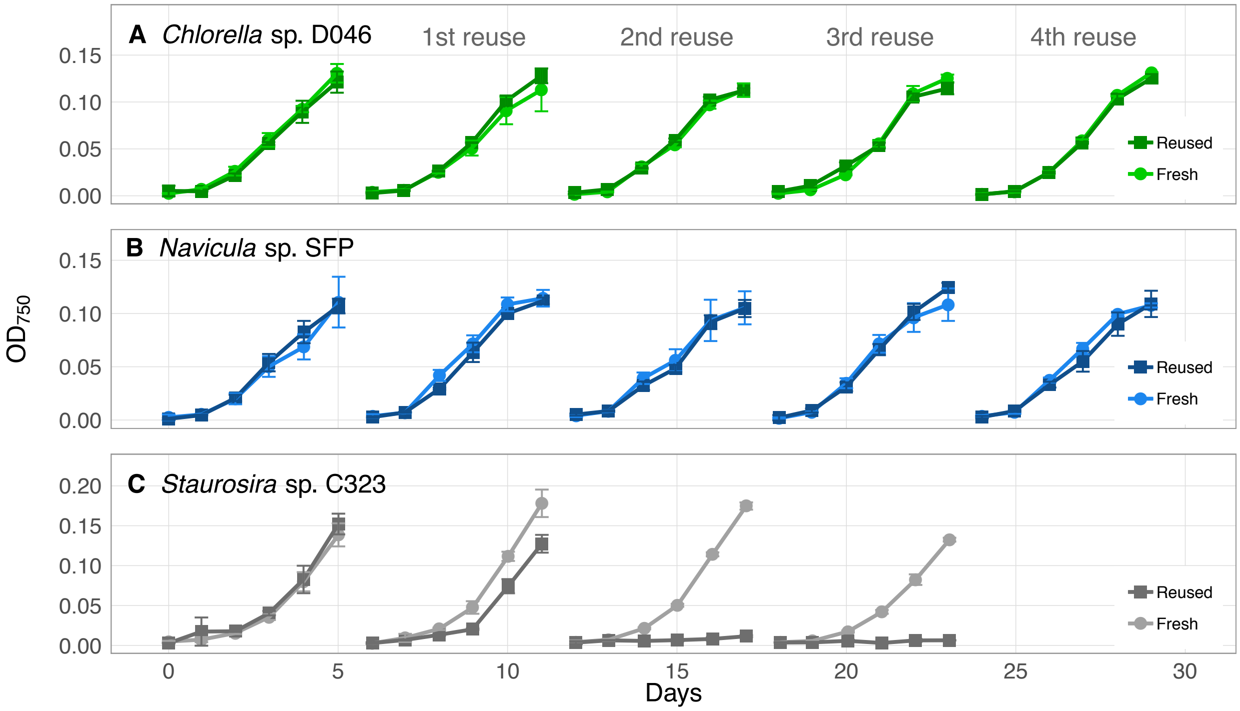

Figure S1: Blank-corrected optical density (OD) at 750 nm of the cultures **(A)** *Chlorella* sp. D046. **(B)** *Navicula* sp. SFP, and **(C)** *Staurosira sp.* C323 in fresh and reused growth medium. Error bars represent standard deviation of three replicate cultures.

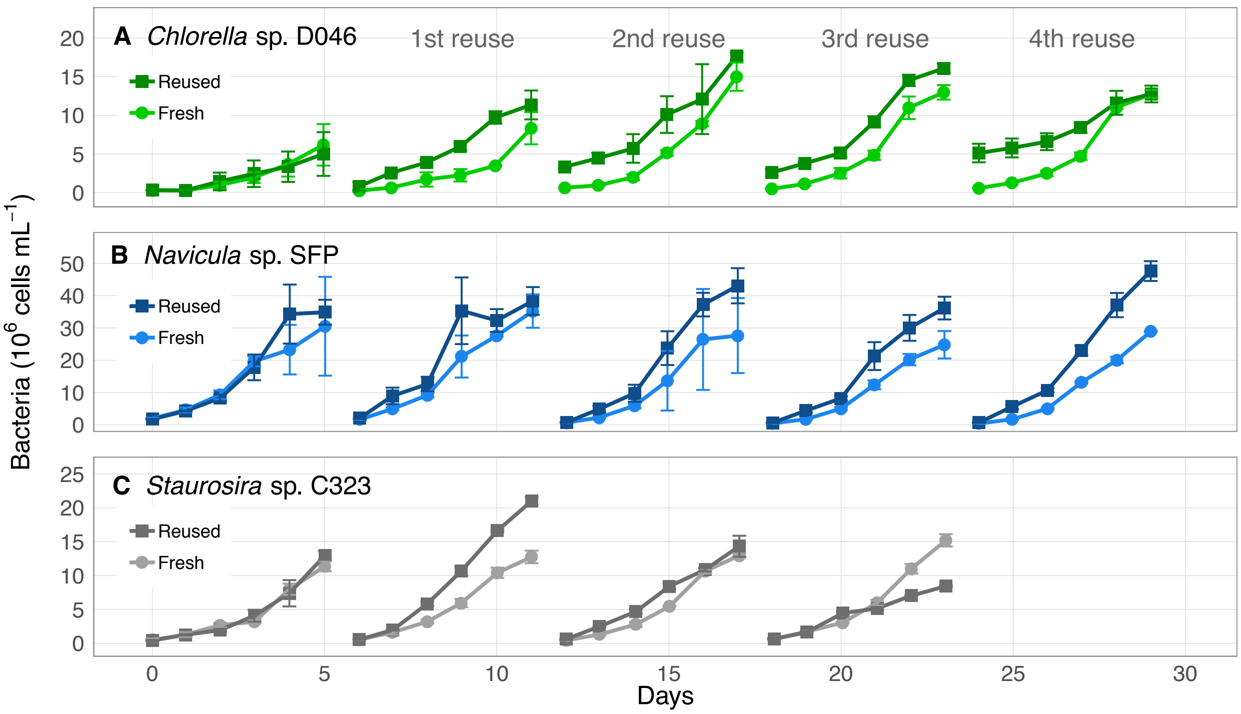

Figure S2: Bacteria cell concentrations over time in cultures of **(A)** *Chlorella* sp. D046, **(B)** *Navicula* sp. SFP, and **(C)** *Staurosira* sp. C323 in fresh and reused growth medium. Error bars represent standard deviation of three replicate cultures.

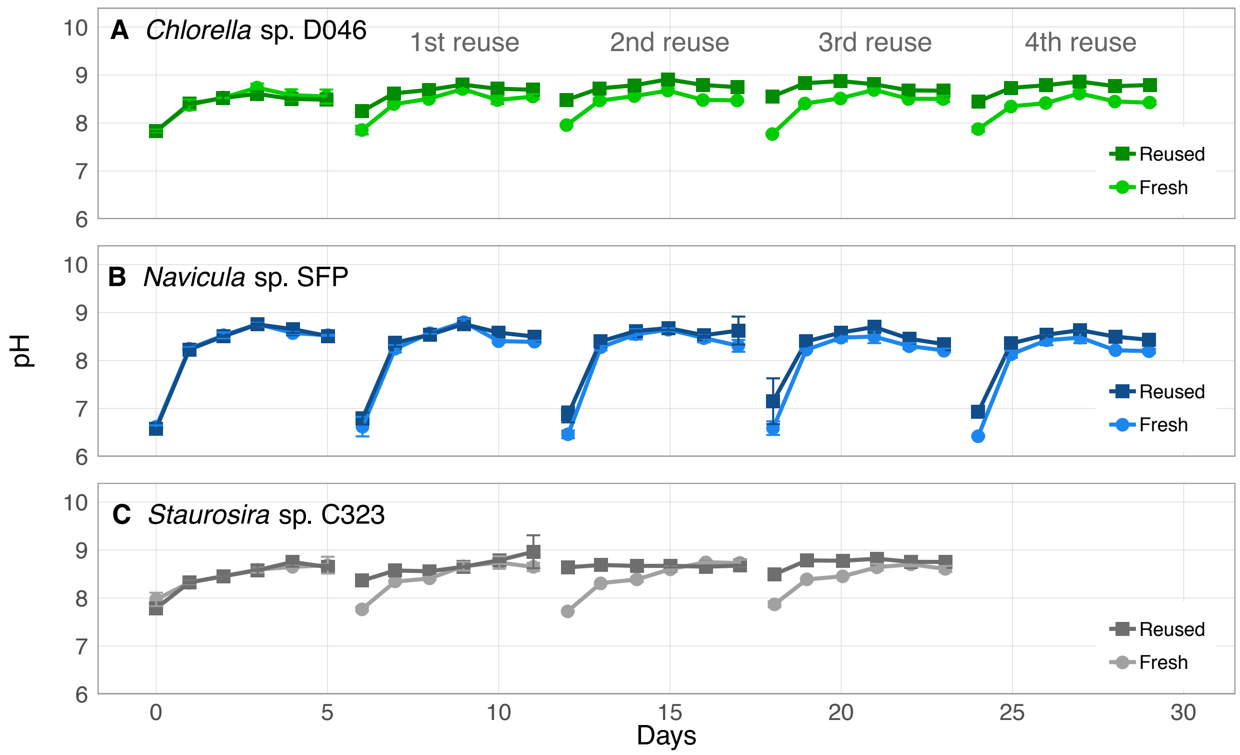

Figure S3: pH of cultures of **(A)** *Chlorella* sp. D046, **(B)** *Navicula* sp. SFP, and **(C)** *Staurosira* sp. C323 in fresh and reused growth medium. Error bars represent standard deviation of three replicate cultures.

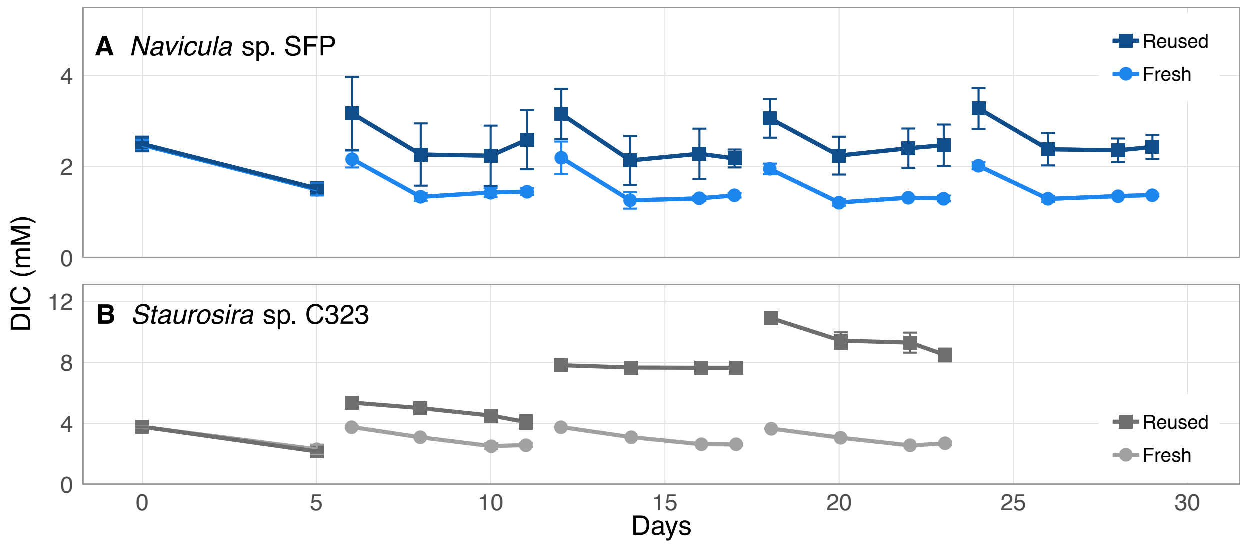

Figure S4: Dissolved inorganic carbon (DIC) in cultures of **(A)** *Navicula* sp. SFP and **(B)** *Staurosira* sp. C323 over time. DIC samples were unavailable for *Chlorella* sp. D046, except for the final day of the experiment in which samples were measured on a Shimadzu TOC-L Analyzer (Fresh Medium = 2.84 ± 0.34 mM, Reused Medium = 8.53 ± 0.29 mM). Error bars represent standard deviation of three replicate cultures.

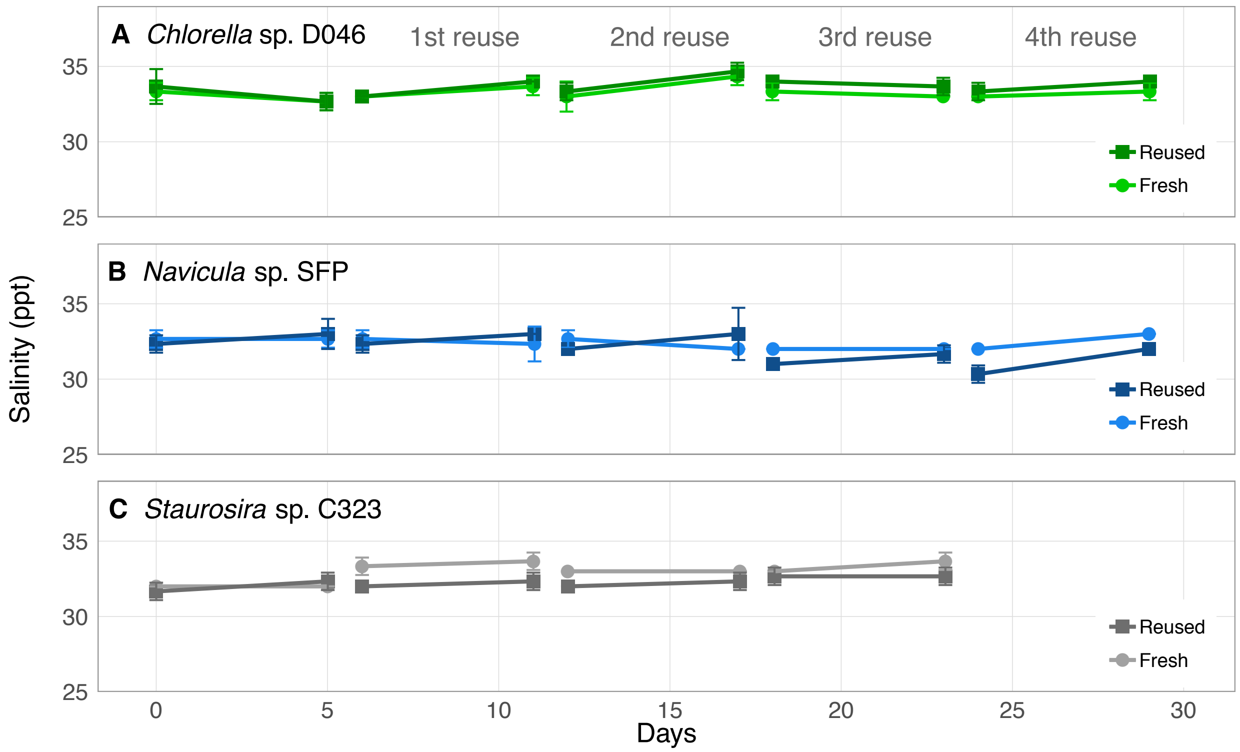

Figure S5: Salinity of cultures of **(A)** *Chlorella* sp. D046, **(B)** *Navicula* sp. SFP, and **(C)** *Staurosira* sp. C323 cultures at the start and end of each round in fresh and reused growth medium. Error bars represent standard deviation of three replicate cultures.

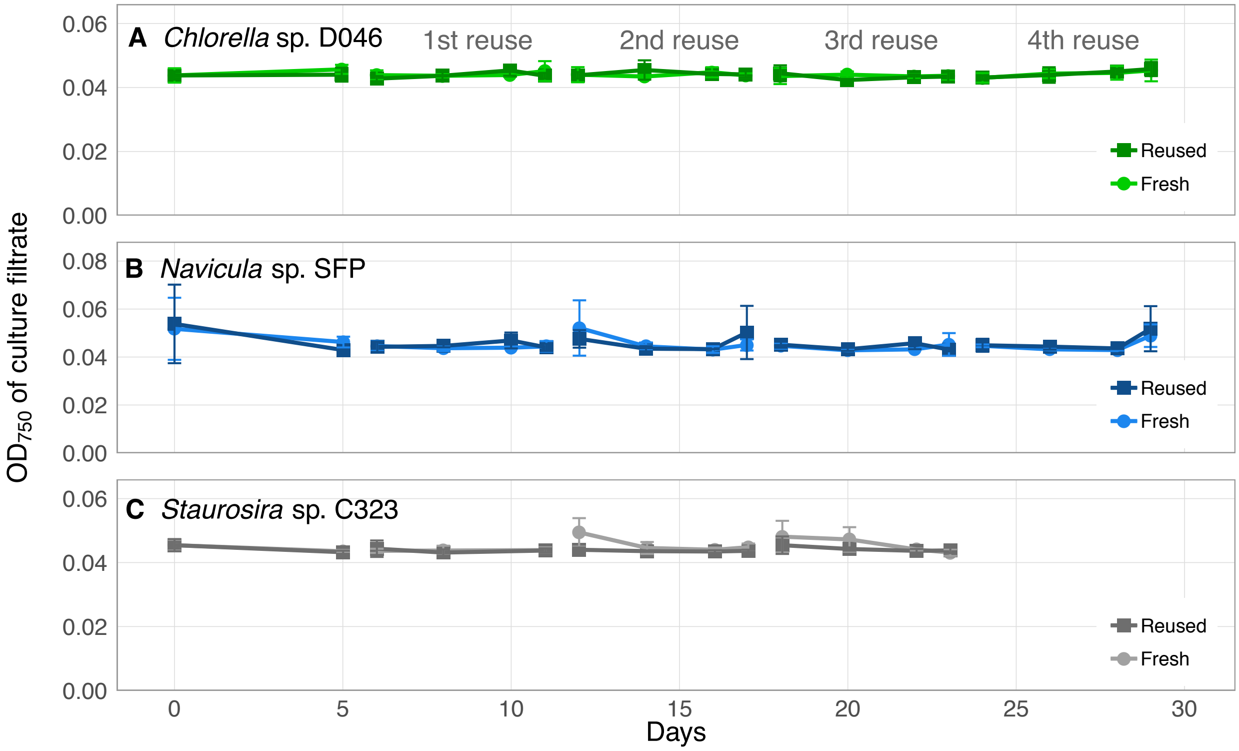

Figure S6: OD at 750 nm of 0.2-µm culture filtrate of **(A)** *Chlorella* sp. D046, **(B)** *Navicula* sp. SFP, and **(C)** *Staurosira* sp. C323 in fresh and reused medium. Error bars represent standard deviation of three replicate culture filtrates. Average OD of ASW medium, measured daily, was 0.0432 ± 0.0009 (n = 84) across all experiments.

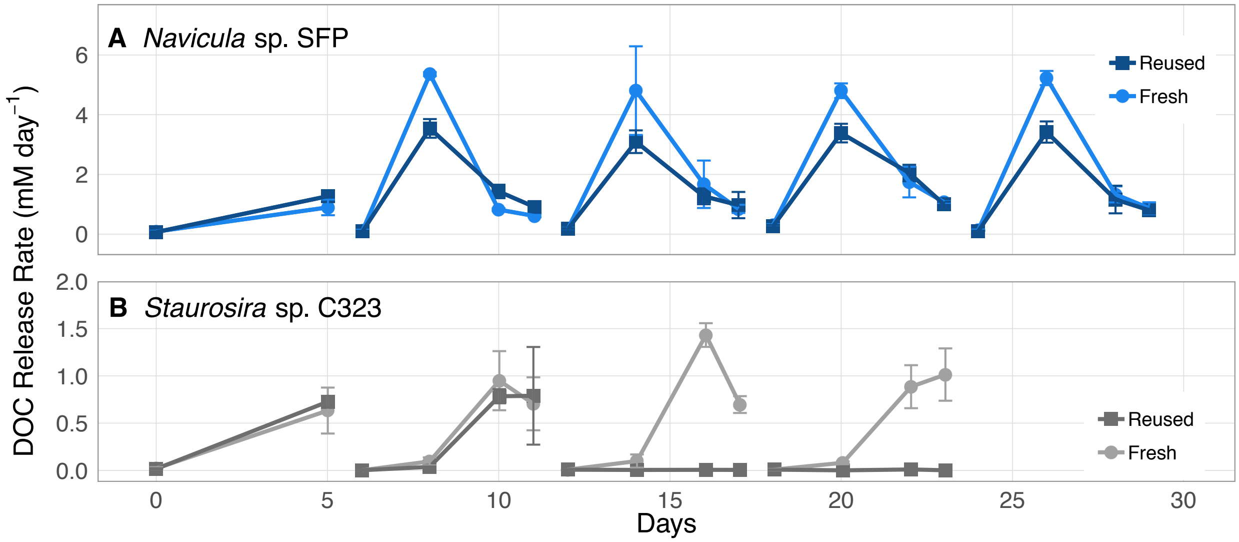

Figure S7: DOC release rates (mM day^{-1}) over time for **(A)** *Navicula* sp. SFP and **(B)** *Staurosira* sp. C323 in fresh and reused growth medium. Error bars represent standard deviation of three replicate cultures.

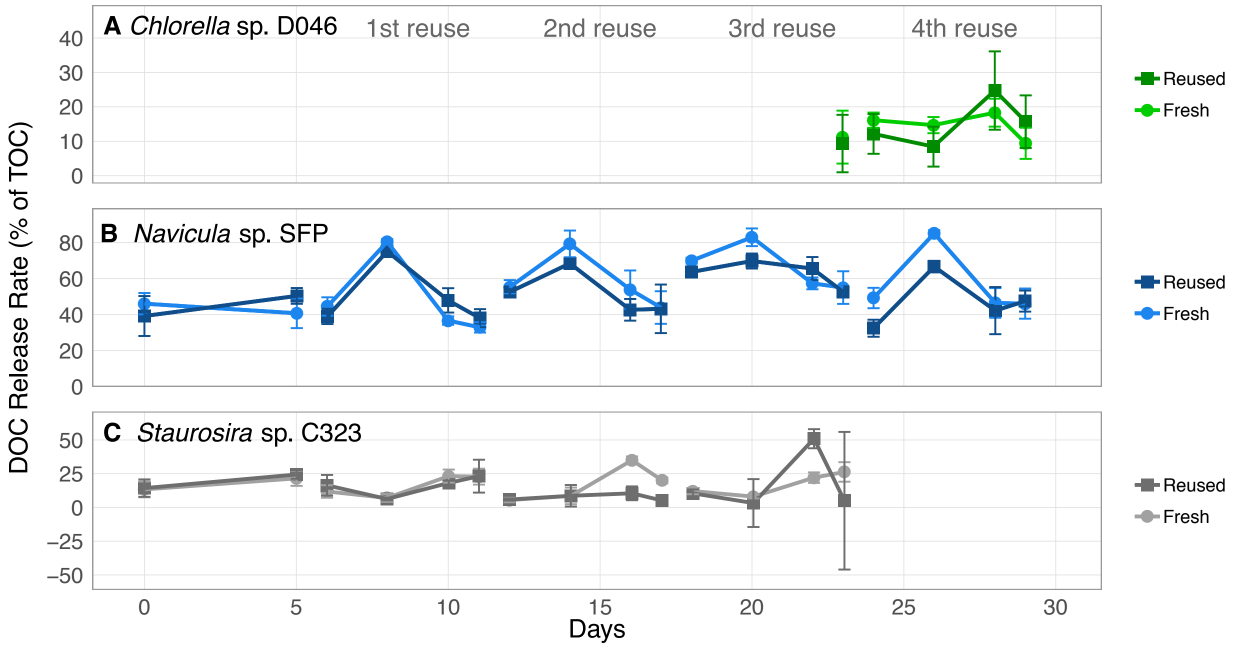

Figure S8: DOC release rate as a percentage of total organic carbon (TOC) production rate over time for **(A)** *Chlorella* sp. D046, **(B)** *Navicula* sp. SFP, and **(C)** *Staurosira* sp. C323 in fresh and reused growth medium. Error bars represent standard deviation of three replicate cultures.

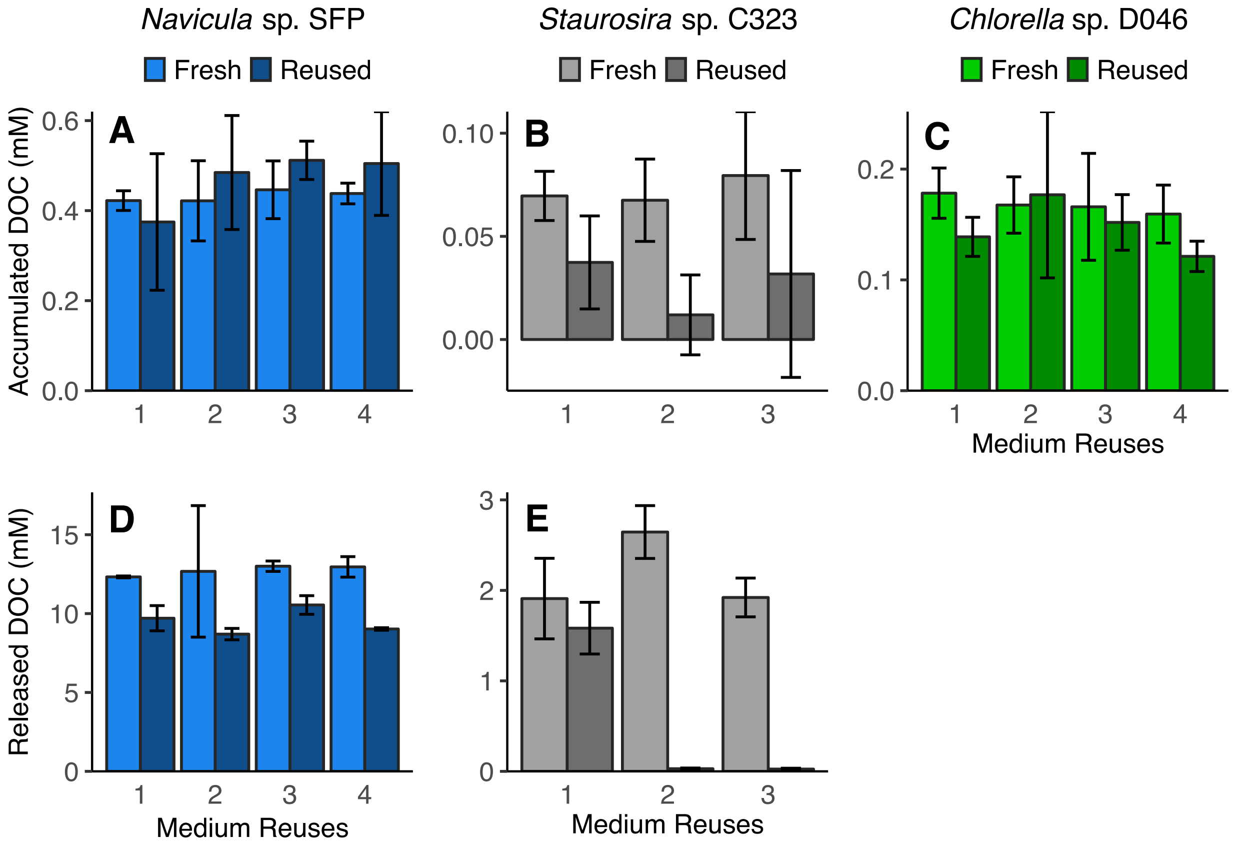

Figure S9: (A-C) Accumulated DOC (difference between Day 0 and Day 5) and **(D, E)** Released DOC (predicted from DOC release rates) per round. Error bars represent standard deviation of three replicate cultures. Note: *Chlorella* sp. D046 Reused Treatment in Reuse 2 is from 2 replicates only. Released DOC data are unavailable for *Chlorella* sp.

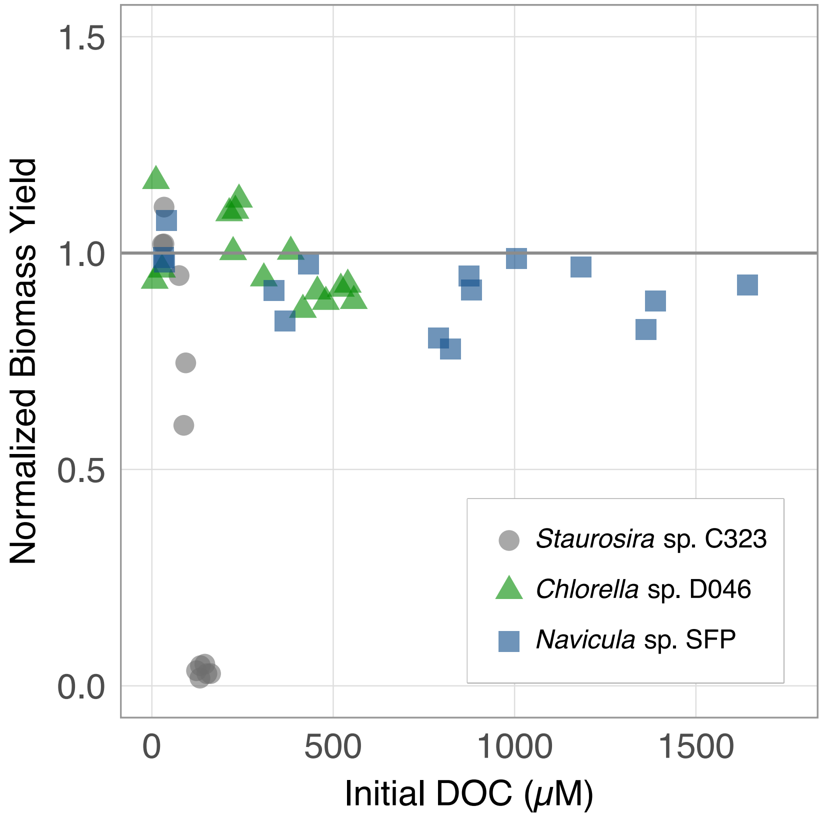

Figure S10: Biomass yield (change in particulate carbon) in reused medium, normalized by mean biomass yield in fresh medium, versus biologically-derived DOC concentration (μM) in reused medium at the start of each round. Bolded horizontal line indicates equal biomass yield in fresh and reused medium.
